# Analgesic Efficacy of Nefopam as an Adjuvant in Patient-Controlled Analgesia for Acute Postoperative Pain After Laparoscopic Colorectal Cancer Surgery

**DOI:** 10.3390/jcm10020270

**Published:** 2021-01-13

**Authors:** Eun Jung Oh, Woo Seog Sim, Won Gook Wi, Jeayoun Kim, Woo Jin Kim, Jin Young Lee

**Affiliations:** Department of Anesthesiology and Pain Medicine, Samsung Medical Center, Sungkyunkwan University School of Medicine, Seoul 06351, Korea; eunjung.oh@samsung.com (E.J.O.); wooseog.sim@samsung.com (W.S.S.); wongook.wi@samsung.com (W.G.W.); jeayoun.kim@samsung.com (J.K.); wj888.kim@samsung.com (W.J.K.)

**Keywords:** colorectal cancer, fentanyl, nefopam, pain, patient-controlled analgesia

## Abstract

Despite rapid advancements in laparoscopic surgical techniques and perioperative management, postoperative pain remains a significant clinical issue. We examined the analgesic efficacy of nefopam as an adjuvant in patient-controlled analgesia (PCA) for acute postoperative pain in patients undergoing laparoscopic colorectal cancer surgery. We retrospectively analyzed the medical records of 120 patients who did or did not receive 80 mg of nefopam as an adjuvant in fentanyl PCA; they were allocated to the nefopam (*n* = 60) or non-nefopam group (*n* = 60). The demographic, clinical, and anesthetic data, with data on pain severity and opioid administration at the postoperative anesthesia care unit (PACU) on postoperative days (PODs) 1, 3, and 5, were compared between the groups. The pain score and opioid administration did not differ at the PACU or on PODs 1, 3, or 5. The day of PCA discontinuation, time to pass flatus, length of the hospital stay, and incidence of nausea/vomiting, dizziness, and headache also did not differ between the groups. Fentanyl PCA with 80 mg of nefopam as an adjuvant did not have a superior analgesic effect after laparoscopic colorectal cancer surgery.

## 1. Introduction

Despite the rapid advancements in laparoscopic surgical techniques and perioperative management, postoperative pain remains a significant clinical issue [1,2,3]. Postoperative pain is insufficiently treated in 30–40% of patients, impacting postoperative recovery, outcome, and quality of life [2,4,5,6,7]. Patient-controlled analgesia (PCA) is commonly used for postoperative pain management. Drugs used for intravenous, epidural, and perineural PCA are based on both opioid and non-opioid analgesics [8]. In a study assessing 39 frequently used drugs for PCA, fentanyl, morphine, and dexmedetomidine are reported to be used most often [8]. Fentanyl is an opioid agonist with approximately 50 to 100 times the potency of morphine [9]. The advantages of fentanyl include a rapid onset and maintenance of cardiovascular stability, while adverse effects include sedation, orthostatic hypotension, nausea, vomiting, bradycardia, as well as central nervous system and respiratory depression [9,10]. In multimodal analgesia, opioid agonists are used as a matrix in combination with non-opioid analgesics; the goal is to reduce the adverse effects caused by a single class of analgesics while enhancing the analgesic effects [11]. Although several analgesic adjuncts to opioid agonists have been studied, including alpha-2 agonist, non-steroidal anti-inflammatory drugs (NSAIDs), and ketamine, it is unclear which combinations are effective [10]. Nefopam exerts antinociceptive and antihyperalgesic effects by influencing monoamines and the noradrenergic and serotonergic systems, resulting in the reduction and prevention of central sensitization [12,13,14]. It has been suggested that the analgesic effect results from reduction in the neurokinin-1 receptor, which may inhibit substance P and neurokinin-1 signaling and subsequently lead to decreased nociceptive responses [12]. Nefopam has been used in several surgical procedures as an analgesic and as a component of multimodal analgesia for enhanced recovery after surgery (ERAS) [15,16,17,18,19]. However, protocols that consider the efficacy and safety, appropriate dose, administration time, administration methods (continuous or bolus injection), and combination with other drugs have not yet been identified. Postoperative pain following colorectal surgery comprises mixed characteristics of somatic, visceral, and neuropathic pain. This is due to the pelvis being narrow, resulting in an increased risk of damage to the autonomic or somatic pelvic nerve plexus during surgery [20,21,22]. This mixture of types of pain requires advanced multimodal analgesia [19]. In this study, we evaluated the analgesic efficacy of nefopam as an adjuvant in fentanyl PCA in patients undergoing laparoscopic surgery for colorectal cancer.

## 2. Materials and Methods

### 2.1. Patients

We retrospectively reviewed the electronic medical records of patients who underwent laparoscopic colorectal surgery from January 2015 to April 2020 at a single center. The patients ranged in age from 27 to 74 years. All patients had tumors within the colon or rectum and had undergone laparoscopic colectomy or anterior resection [23]. The exclusion criteria were as follows: patients over 75 years; cases of emergency surgery; metastatic colorectal lesions; conversion to open surgery; unstable vital signs during anesthesia; a lack of follow-up data; intensive care unit management after surgery; the inability to express pain severity; the requirement for a PCA pump refill after completion of the first PCA reservoir; postoperative complications including ileus, anastomotic leak, hemorrhage, or wound infection; patients receiving analgesics (acetaminophen, NSAIDs, tramadol, or glucocorticoids) during anesthesia; and nefopam contraindications [23,24]. Nefopam contraindications included preoperative tachycardia (>100 beats per min), uncontrolled arrhythmia, unstable angina, symptomatic congestive heart failure, hepatic or renal insufficiency, and cognitive impairments [24]. This study was approved by the Institutional Review Board of Samsung Medical Center (SMC 2020-05-009) and registered with the Clinical Research Information Service of the Korea National Institute of Health (http://cris.nih.go.kr/cris/index.jsp, KCT0005037). The need for individual consent was waived by the institutional review board, as this was a retrospective study involving medical record review.

### 2.2. Anesthesia

Patients did not receive premedication before surgery. After admission to the operating room, standard monitoring (IntelliVue MP70, Philips Healthcare, Best, Netherlands) was performed, including oxygen saturation, electrocardiography, end-tidal carbon dioxide, pulse oximetry, bispectral index (BIS), and non-invasive blood pressure. Anesthesia was induced intravenously using 40 mg of 2% lidocaine, 2 mg/kg of 2% propofol, 0.5–1 µg/kg of fentanyl, 0.05 mg/kg of midazolam, and 0.6–0.8 mg/kg of rocuronium. The patient was endotracheally intubated using a Macintosh laryngoscope after approximately 3–5 min of mask ventilation and the loss of all four twitches upon train-of-four ulnar nerve stimulation. Following tracheal intubation, anesthesia was maintained using 1.5–3.0 vol% sevoflurane and a bolus injection of 0.5–1 µg/kg fentanyl to maintain hemodynamic parameters within 20% of baseline values and a BIS of 40–60. The lungs were ventilated with 50% oxygen with air. This was adjusted to maintain an end-tidal carbon dioxide level of 30–40 mmHg. All surgeries were performed by one of six specialized colorectal surgeons who followed similar techniques for colorectal cancer [20]. At the time of skin closure, patients received an intravenous PCA pump (Automed 3200^®^, Ace Medical, Seoul, Korea) with a basal infusion rate of 0.5 mL/h, a bolus of 1 mL, and a lockout interval of 15 min. The drugs used for PCA comprised a 100 mL mixture of 20 µg/kg fentanyl in normal saline, whereas the nefopam addition to PCA comprised a 100 mL mixture of 80 mg nefopam (Acupan^®^, Pharmbio Korea, Seoul, Korea) and 20 µg/kg fentanyl in normal saline. At the end of surgery, patients were administered 4 mg/kg of intravenous sugammadex and/or 0.03 mg/kg of intravenous pyridostigmine, and 0.002 mg/kg of intravenous glycopyrrolate. The reversal agents were decided by the anesthesiologists considering the neuromuscular block status. After extubation, patients were moved to the postoperative anesthesia care unit (PACU) where they received a further bolus of intravenous fentanyl at 0.5 µg/kg when the numeric rating scale (0 = no pain to 10 = absolutely intolerable pain) score was greater than 3. Postoperative opioids at the general ward included intravenous fentanyl, meperidine, or hydromorphone, as well as oral medications, including oxycodone and tapentadol [25]. Opioid administration was recorded by conversion to fentanyl units on postoperative days (PODs) 1, 3, and 5 [25]. Pain scores were recorded upon arrival at the PACU and at discharge from the PACU, as well as on PODs 1, 3, and 5.

### 2.3. Statistical Analysis

All data were analyzed using SAS 9.4 (SAS Institute, Cary, NC, USA). Data are expressed as the mean ± standard deviation (SD) or number (proportion), as appropriate. Demographic, clinical, and anesthetic data for the two groups were compared using a chi-squared test, *t*-test, or Fisher’s exact test. We extracted the mean arterial pressure (MAP), heart rate, and BIS before intubation (T1), at the start of surgery (T2), at the end of surgery (T3), and after extubation (T4); these parameters were compared using the *t*-test or Wilcoxon rank-sum test. We compared the pain scores at PACU arrival and discharge, as well as on PODs 1, 3, and 5 with rest and movement. Moreover, we compared the opioid administration during anesthesia, at the PACU, and on PODs 1, 3, and 5 using the Wilcoxon rank-sum test. The chi-squared test was used to compare the day of PCA discontinuation, time to pass flatus, length of the hospital stay, and the postoperative incidence of nausea/vomiting, dizziness, and headache. A *p*-value less than 0.05 was considered statistically significant.

## 3. Results

Of the 237 patients who were assessed for eligibility, 117 were excluded based on the exclusion criteria. Thus, data of 120 patients were analyzed. The patients were categorized into two groups, namely those with PCA containing 80 mg of nefopam (nefopam group, *n* = 60) and those with PCA that did not contain nefopam (non-nefopam group, *n* = 60). Demographic and clinical data are summarized in Table 1. Age, sex, body mass index, American Society of Anesthesiologists (ASA) physical status classification, diagnosis, surgery type, pathological stage, maximum tumor size, ileostomy, anesthesia time, preoperative pain, and rocuronium dose were not significantly different between the groups (Table 1). Intraoperative fentanyl administration was higher in the nefopam group. The MAP at T1 and T2 was higher in the nefopam group, which may have led to the higher fentanyl administration to control MAP during anesthesia (Table 2). No difference was found regarding heart rate or BIS between the groups. There were no significant differences in the postoperative pain across the time points from admission to the PACU to POD 5 (Table 3). Additionally, no differences were found regarding the fentanyl dose used in PCA, fentanyl dose from the PACU to POD 5, PCA discontinuation day, time to pass flatus, or length of hospital stay (Table 4). The postoperative incidences of nausea/vomiting, dizziness, headache, and somnolence were also not different between the groups (Table 5).

All data are presented as the mean ± SD or number (%) of patients. ASA, American Society of Anesthesiologists; NRS, numeric rating scale; Nefopam group, patients who received nefopam as an adjuvant in patient-controlled analgesia; Non-nefopam group, patients who did not receive nefopam as an adjuvant in patient-controlled analgesia. * *p*-value < 0.05 was considered statistically significant.

All data are presented as the mean ± SD. T1, before intubation; T2, start of surgery; T3, end of surgery; T4, after extubation; Nefopam group, patients who received nefopam as an adjuvant in patient-controlled analgesia; Non-nefopam group, patients who did not receive nefopam as an adjuvant in patient-controlled analgesia. * *p*-value < 0.05 was considered statistically significant.

All data are presented as the mean ± SD. NRS, numeric rating scale; PACU, postoperative anesthesia care unit; POD, postoperative day; Nefopam group, patients who received nefopam as an adjuvant in patient-controlled analgesia; Non-nefopam group, patients who did not receive nefopam as an adjuvant in patient-controlled analgesia. *p*-value < 0.05 was considered statistically significant.

All data are presented as the mean ± SD. PACU, postoperative anesthesia care unit, POD, postoperative day; Nefopam group, patients who received nefopam as an adjuvant in patient-controlled analgesia; Non-nefopam group, patients who did not receive nefopam as an adjuvant in patient-controlled analgesia. *p*-value < 0.05 was considered statistically significant.

All data are presented as number (%) of patients. Nefopam group, patients who received nefopam as an adjuvant in patient-controlled analgesia; Non-nefopam group, patients who did not receive nefopam as an adjuvant in patient-controlled analgesia. *p*-value < 0.05 was considered statistically significant.

## 4. Discussion

In the present study, we aimed to evaluate whether nefopam as an adjuvant in fentanyl PCA exerts a superior analgesic effect in patients undergoing laparoscopic colorectal cancer surgery. Postoperative pain severity and opioid administration were not reduced during the acute postoperative period and neither was the incidence of postoperative nausea/vomiting, dizziness, headache, or somnolence. We suspect that these results are related to the low nefopam dose for prolonged analgesia, and/or the timing of nefopam administration for preemptive analgesia.

The ERAS protocols in surgery for colorectal cancer are designed to improve patient outcomes [26,27,28]. Early recovery of bowel function is enhanced by a reduction of opioids [29]. The guidelines recommend a multimodal approach to pain control, which is based on the combined use of opioids and opioid-sparing analgesics, such as N-methyl-D-aspartate (NMDA) antagonist, NSAIDs, acetaminophen, gabapentinoids, glucocorticoids, and tramadol [30]. However, these non-opioid analgesics have been shown to be limited in their safety or efficacy [31]. Postoperative NSAIDs following colorectal surgery showed improved pain score, opioid sparing effect, and enhanced early recovery of bowel function, but NSAIDs increased anastomotic leak by inhibiting the normal wound healing process and interfering with angiogenesis [31]. Perioperative glucocorticoid following cancer surgery produces analgesic effects at a high dose, but high and repetitive doses might have immunosuppressive effects [32]. NMDA antagonists are pharmacological agents used in central sensitization and neuropathic pain [33]. The NMDA class of glutamate receptors is involved in pain transmission and plasticity or enhancement [34]. Ketamine, as an NMDA antagonist, results in decreased perioperative pain and opioid use, and has antihyperalgesic, antiallodynic, and antitolerance properties. However, in colorectal surgery, the efficacy of ketamine is insufficient [35]. Nefopam exerts an analgesic effect by indirectly modulating NMDA receptors [36]; this effect has been reported for acute and chronic pain, alone and in combination with other drugs [37]. A comparison of intraoperative nefopam and ketamine infusion showed intraoperative opioid reduction, and nefopam showed superior properties for pain reduction and has been preferred for reduced sedation [38]. A 20 mg dose of nefopam has an analgesic potency equal to 6–12 mg of morphine or 50 mg of meperidine [39]. When nefopam was first introduced as an analgesic, the median effective dose (ED50) for surgery ranged from 17 to 28 mg [40]. The analgesia onset time is at least 15 min and the peak analgesic effect occurs 30–60 min post-infusion [41]. Kim and colleagues [41] reported that the ED50 of nefopam was 62.1 mg for pain related to laparoscopic cholecystectomy. The adverse effects of nefopam include pain at the injection site upon administration of a higher dose, nausea, vomiting, somnolence, dizziness, hyperhidrosis, headache, and seizure, while neurological adverse effects are typical in nefopam overdose [36,41,42,43]. The daily maximum recommended intravenous dosage of nefopam in healthy individuals is 20 mg every 4 h [43]. Therefore, proper dosage of nefopam between clinical benefits and adverse effects should be adjusted based on the surgery type and the patient’s status. In renal transplantation, the simultaneous administration of 160 mg of nefopam with fentanyl PCA had a 19% fentanyl-sparing effect during 48 h of follow-up [24]. An addition of 200 mg and 400 mg of nefopam to fentanyl PCA reduced fentanyl consumption from 48.9% to 54.5% during 48 h of follow-up after laparoscopic hysterectomy [16]. In our study, we infused a mixture containing 80 mg of nefopam in the fentanyl PCA, which resulted in no additional analgesic efficacy, but also no adverse effects.

Several studies have reported on the timing of nefopam administration for postoperative analgesia. Preoperative administration of 20 mg of nefopam reduced exertional pain during 2 to 72 h of follow-up after colon cancer surgery, although it did not reduce postoperative fentanyl consumption [15]. Furthermore, intraoperative administration of 40 mg of nefopam decreased pain at 6 h after laparoscopic gastrectomy, although it did not decrease pain after 6 h [17]. Postoperative administration of 20 mg of nefopam at the PACU did not reduce pain during 48 h of follow-up after arthroscopic rotator cuff surgery [18]. In our study, we initiated PCA administration at the time of skin closure. We suspect that a combination of preemptive nefopam administration and a larger dose used in the PCA may lead to a superior analgesic efficacy. Further studies regarding nefopam protocols are needed to determine the appropriate dose, timing, and duration of administration.

This study has several limitations. First, we did not convert the postoperatively administered non-opioid analgesics (acetaminophen, NSAIDs, or tramadol) into opioid equivalents because of conversion difficulties, although it can affect postoperative pain. Additionally, we did not enroll patients who had taken non-opioid analgesics during anesthesia. These exclusions negatively affect the generalization of the usual perioperative experience. Second, we did not measure the daily delivered and rescue bolus doses of PCA. Third, the sample size of our study was small. Fourth, we did not use propensity score matching. To minimize selection bias, we applied the exclusion criteria of nefopam for patients in the non-nefopam group. However, there may have been possible unknown confounding factors in the selection of patients in the non-nefopam group.

## 5. Conclusions

We observed no superior analgesic effects using 80 mg of nefopam as an adjuvant in fentanyl PCA during the acute postoperative period. Further randomized controlled studies are needed to determine an effective and safe dose and administration regimen for nefopam to be used as an adjuvant to fentanyl or other opioids in advanced multimodal analgesia for PCA.

## Figures and Tables

**Table 1:** Demographic and clinical data.

	All Patients	Nefopam Group	Non-Nefopam Group	*p*-Value
	(*n* = 120)	(*n* = 60)	(*n* = 60)	
Age (y)	58.9 ± 10.7	59.0 ± 11.3	58.8 ± 10.0	0.848
Sex (M/F)	60/60	30/30	30/30	>0.999
Body mass index (kg/m^2^)	23.9 ± 3.0	24.0 ± 2.7	23.9 ± 3.2	0.715
ASA status: I/II/III	62/56/2	27/33/0	35/23/2	0.071
Diagnosis				0.568
Colon cancer	77 (64.2%)	37 (61.7%)	40 (66.7%)	
Rectal cancer	43 (38.8%)	23 (38.3%)	20 (33.3%)	
Surgery type				0.838
Colectomy	33 (27.5%)	16 (26.7%)	17 (28.3%)	
Anterior resection	87 (72.5%)	44 (73.3%)	43 (71.7%)	
Pathological stage 0/1/2/3/4	19/8/21/22/50	7/3/10/12/28	12/5/11/10/22	0.604
Maximum tumor size >4 cm	42 (35.0%)	24 (40.0%)	18 (30.0%)	0.251
Ileostomy	5 (4.2%)	3 (5.0%)	2 (3.3%)	>0.999
Anesthesia time (min)	188.7 ± 53.7	196.7 ± 56.6	180.6 ± 49.8	0.103
Preoperative pain (NRS)	0.0 ± 0.0	0.0 ± 0.0	0.0 ± 0.0	1.000
Rocuronium dose (mg)	75.6 ± 17.6	76.2 ± 18.6	75.0 ± 16.6	0.979
Intraoperative fentanyl (µg)	73.1 ± 27.2	78.1 ± 27.9 *	68.1 ± 25.7	0.044

*, there was significant difference between the groups.

**Table 2:** Intraoperative vital signs and bispectral index (BIS) values over time.

	All Patients	Nefopam Group	Non-Nefopam Group	*p*-Value
	(*n* = 120)	(*n* = 60)	(*n* = 60)	
Mean Arterial Pressure				
T1	93.3 ± 9.8	95.5 ± 10.5 *	91.2 ± 8.7	0.017
T2	86.5 ± 18.9	93.5 ± 16.4 *	79.6 ± 18.8	<0.001
T3	85.5 ± 12.9	84.7 ± 12.1	86.3 ± 13.7	0.278
T4	96.1 ± 15.4	97.3 ± 13.5	94.9 ± 17.2	0.390
Heart Rate				
T1	75.9 ± 11.8	75.5 ± 12.4	76.3 ± 11.3	0.567
T2	79.2 ± 13.5	80.2 ± 12.9	78.1 ± 14.2	0.391
T3	69.8 ± 11.3	68.9 ± 10.7	70.8 ± 12.0	0.315
T4	81.5 ± 12.5	80.5 ± 11.2	82.4 ± 13.8	0.405
Bispectral Index				
T1	94.0 ± 7.5	95.0 ± 3.5	93.1 ± 9.9	0.234
T2	38.0 ± 7.1	38.2 ± 6.6	37.8 ± 7.6	0.760
T3	52.2 ± 9.3	51.3 ± 9.0	53.0 ± 9.5	0.362
T4	81.1 ± 9.1	82.7 ± 6.4	79.5 ± 10.9	0.064

*, there was significant difference between the groups.

**Table 3:** Postoperative pain scores.

Postoperative Pain (NRS)	All Patients (*n* = 120)	Nefopam Group (*n* = 60)	Non-Nefopam Group (*n* = 60)	*p*-Value
PACU				
At admission	6.6 ± 1.7	6.6 ± 1.7	6.6 ± 1.7	0.919
At discharge	2.9 ± 1.0	3.0 ± 0.6	2.8 ± 0.6	0.467
POD 1				
Rest	2.9 ± 0.3	2.9 ± 0.3	3.0 ± 0.2	0.470
Movement	4.4 ± 1.7	4.5 ± 1.7	4.4 ± 1.8	0.478
POD 3				
Rest	2.9 ± 0.3	3.0 ± 0.3	2.9 ± 0.4	0.200
Movement	3.8 ± 1.4	3.6 ± 1.3	3.9 ± 1.5	0.230
POD 5				
Rest	2.9 ± 0.4	2.9 ± 0.4	2.9 ± 0.4	0.641
Movement	3.1 ± 0.9	3.0 ± 0.7	3.1 ± 1.0	0.618

**Table 4:** Postoperative data.

	All Patients	Nefopam Group	Non-Nefopam Group	*p*-Value
	(*n* = 120)	(*n* = 60)	(*n* = 60)	
Fentanyl dose for PCA (µg)	1260.0 ± 201.0	1228.3 ± 194.9	1291.7 ± 203.6	0.104
Fentanyl dose at PACU (µg)	49.7 ± 27.7	49.3 ± 26.7	50.2 ± 28.9	0.830
Fentanyl dose post-surgery (µg)				
POD 1	64.5 ± 82.0	69.2 ± 86.1	59.7 ± 78.1	0.666
POD 3	136.8 ± 91.0	133.4 ± 92.7	140.1 ± 90.0	0.587
POD 5	171.1 ± 70.0	175.0 ± 68.0	167.3 ± 72.2	0.581
PCA discontinuation day (POD)	2.4 ± 0.9	2.4 ± 1.0	2.4 ± 0.8	0.751
Time to pass flatus (POD)	2.8 ± 0.9	2.9 ± 0.9	2.7 ± 0.9	0.101
Length of hospital stay (POD)	6.2 ± 1.0	6.2 ± 1.1	6.1 ± 0.1	0.592

**Table 5:** Postoperative complications.

	All Patients	Nefopam Group	Non-Nefopam Group	*p*-Value
	(*n* = 120)	(*n* = 60)	(*n* = 60)	
Nausea/vomiting	25 (20.8%)	9 (15.0%)	16 (26.7%)	0.116
Dizziness	6 (5.0%)	2 (3.3%)	4 (6.7%)	0.679
Headache	7 (5.8%)	3 (5.0%)	4 (6.7%)	>0.999
Somnolence	2 (1.7%)	1 (1.7%)	1 (1.7%)	>0.999

## Data Availability

The data presented in this study are available on request from the corresponding author. The data are not publicly available due to ethical reason.
